# Understanding the uptake of a national retinopathy screening programme: An audit of people with diabetes in two large primary care centres

**DOI:** 10.12688/hrbopenres.12926.2

**Published:** 2019-11-27

**Authors:** Marsha Tracey, Emmy Racine, Fiona Riordan, Sheena M. McHugh, Patricia M. Kearney

**Affiliations:** 1 AXIS Healthcare Consulting Ltd, Dublin, Ireland; 2 School of Public Health, University College Cork, Cork, Cork, T12 XF62, Ireland

**Keywords:** diabetes, retinopathy, screening, microvascular complication, primary care

## Abstract

**Background: **Diabetic retinopathy (DR) affects 8.2% of the Irish population with type 2 diabetes over 50 years and is one of the leading causes of blindness among working-age adults. Regular diabetic retinopathy screening (DRS) can reduce the risk of sight loss. In 2013, the new national screening programme (RetinaScreen) was introduced in Ireland. Maximising DRS uptake (consent to participate in the programme *and* attendance once invited) is a priority, therefore it is important to identify characteristics which determine DRS uptake among those with diabetes in Ireland. We report uptake in an Irish primary care population during the initial phase of implementation of RetinaScreen and investigate factors which predict consenting to participate in the programme.

**Methods:** In two primary care practices, data were extracted from records of people with diabetes (type 1 and type 2) aged ≥18 years who were eligible to participate in RetinaScreen between November 2013 and August 2015. Records were checked for a RetinaScreen letter. RetinaScreen were contacted to establish the status of those without a letter on file. Multivariable Poisson regression was used to examine associations between socio-demographic variables and consenting. Adjusted incident rate ratios (IRR) with 95% CI were generated as a measure of association.

**Results:** Of 722 people with diabetes, one fifth (n=141) were not registered with RetinaScreen. Of 582 who were registered, 63% (n=365) had participated in screening. Most people who consented subsequently attended (n=365/382, 96%). People who had attended another retinopathy screening service were less likely to consent (IRR 0.65 [95%CI 0.5-0.8]; p<0.001). Other predictors were not significantly associated with consent.

**Conclusions:** Over one third of eligible participants in RetinaScreen had not consented. Research is needed to understand barriers and enablers of DRS uptake in the Irish context. Implementing strategies to improve DRS uptake, barriers to consent in particular, should be a priority.

## Introduction

Diabetic retinopathy (DR) is the most common microvascular complication of diabetes. DR affects 8.2% of the Irish population over 50 years with type 2 diabetes ^1^ and is the leading causes of blindness among adults of working age ^2^. This estimate is based on self-report; studies among regional cohorts of primary care patients with type 1 and 2, based on objective data, have reported higher estimates (25–26%) ^3, 4^. Regular diabetic retinopathy screening (DRS) leads to the earlier detection of retinopathy and treatment that can prevent or delay the development of diabetes-related blindness. Although DRS is found to be effective, few countries have established a population-based DRS programme. In 2013, the new national programme (Diabetic RetinaScreen) was introduced in Ireland offering free, regular retinopathy screening to people with diabetes.

Ensuring a high uptake of retinopathy screening is challenging ^5^. Prior to the introduction of a national programme, there was variation in attendance at regional screening services in Ireland, with attendance rates ranging from 49–80% ^3, 4, 6, 7^. Screening uptake has also been identified as a challenge internationally; with attendance rates ranging from 28–92% ^8– 14^. Non-attendance at screening has been identified as a risk factor for poor visual outcomes among those with diabetes ^15^. Factors associated with non-attendance include, younger age ^9, 11^, type 1 diabetes ^9^, poor glycaemic control ^9, 16^ and lack of awareness of the benefits of DRS or the risk of DR among people with diabetes ^6, 17^. A recommendation from a healthcare provider ^6, 17^ and fear of impaired vision ^17^ have been shown to motivate attendance. Little is known about characteristics which determine the uptake of retinal screening among those with diabetes in the Irish context ^4, 6^. The aim of this study was to identify factors associated with participation in a new national retinopathy screening service using data from primary care.

## Methods

### National Screening Programme

**Figure 1.**
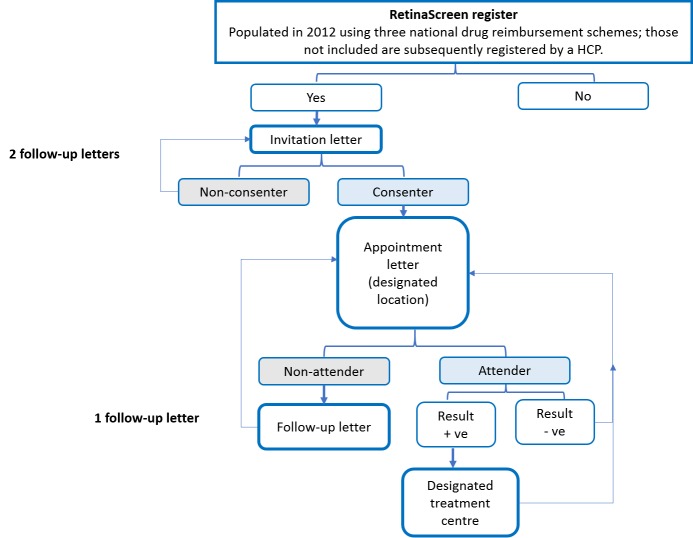
Flow diagram illustrating process of consenting and attending to the programme. HCP; Health Care Professional.

RetinaScreen is a government-funded programme providing free, annual retinal screening, and, if necessary, treatment, to anyone aged 12 years or older with diagnosed diabetes. The programme was commissioned in 2011 and rolled out in 2013 and 2014 ^18, 19^. The current study was conducted during the initial phase of the programme (2013–2015). In Ireland, there is no national register of people with diabetes. The programme register was populated in 2012 using information from existing national health schemes, specifically pharmacy claims data. GPs or by other healthcare professionals involved in diabetes care can also add people with diabetes to the register by directly contacting RetinaScreen. All those on the register are invited by letter to participate in the programme ^18, 19^, after which they provide consent for the programme to hold and use their contact details and receive an appointment. Once consented they receive an appointment for a fixed time in their local screening centre. They can contact RetinaScreen to change the time and date of their appointment. Once consented they receive an appointment, after which they need to attend. Figure 1 illustrates this process of registration, consenting to and attending the programme. 

### Population

Members of the target population were people with diabetes aged 18 years and over who were eligible to participate in RetinaScreen during the uptake period of interest, that is, diagnosed with diabetes four months before the end of the uptake period of interest (Practice A: between July 2014 and August 2015; Practice B between November 2013 and December 2014).

### Research setting

**Figure 2.**
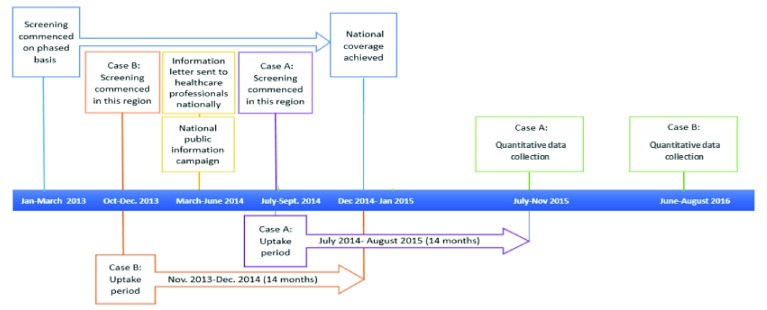
Timeline of the national programme, RetinaScreen, and data collection at study sites.

Data collection was carried out across two large primary healthcare centres (Practice A and Practice B) located in two different Community Health Organisations in Ireland ( Figure 2). Practice A had seven GPs with five practice nurses and approximately 22,000 patients. Practice B had eight GPs with four practice nurses and approximately 20,000 patients.

### Data collection

The two primary care centres used the same computerised IT system, therefore data collection methods described were carried out across both sites. All adults aged ≥18 years with diabetes were identified via the practice database, using the International Classification of Primary Care, Second Edition (ICPC-2) codes for diabetes insulin dependent (T89) and diabetes non-insulin dependent (T90). Duplicates were removed and data were extracted from each individual medical record. Next, the following inclusion criteria were applied: age 18 years and older, community-dwelling, diagnosed with diabetes at least four months before the end of the uptake period of interest (Practice A: before May 2015; Practice B: before September 2014). Exclusion criteria were, a diagnosis of prediabetes or gestational diabetes or diabetes insipidus, no perception of light in both eyes (blindness) as documented in medical records, nursing home residence, visiting patient to the practice.

Data were extracted from eligible individual’s medical record. Each medical record was checked for a RetinaScreen letter (results letter or did not attend letter). RetinaScreen was contacted to establish the status of those who did not have a letter on file. Individuals were then categorised into four groups:

1. Not registered (details were not listed on the RetinaScreen database),
2. Non-consenters (details were listed in the RetinaScreen database but did not respond to the RetinaScreen initial letter asking for individual’s consent to hold and use their contact details),
3. Non-attenders (details were listed on the RetinaScreen database; they responded to the RetinaScreen invitation letter but did not attend screening appointment)
4. Attenders (details were listed on the RetinaScreen database, responded to the RetinaScreen invitation letter and attended appointment).

In each practice, the beginning of the uptake period was defined as the earliest date of the first screening results letter available on file ( Figure 2). The end of the uptake period was defined as the last day of data collection; hence the uptake period for each practice was 14 months in duration. 

Individual-level characteristics were also extracted from the patient’s medical records and included: date of birth, gender, healthcare cover (medical card/private insurance), diabetes type (type 1/type 2), date of GP diabetes diagnosis (≤2012 vs. >2012) and a previous doctor diagnosis of hypertension. A previous diagnosis of myocardial infarction, congestive cardiac failure, cerebrovascular accident and transient ischaemic attack were defined as macrovascular complications. A previous diagnosis of DR, diabetic neuropathy or diabetic nephropathy were defined as microvascular complications. Each medical record was checked for a results letter from existing retinopathy screening services; attendance at existing retinopathy screening services (for example a private ophthalmologist or previous regional initiative) was categorised into two groups: no evidence of attending existing retinopathy screening services (‘none’) and evidence of attending existing retinopathy screening services (‘previous attendance’). Age (years) was calculated by subtracting year of birth from year of uptake period and was categorised into three age groups (18–39 years; 40–65 years; 65 years and over). Duration of diabetes diagnosis was calculated by subtracting year of GP diabetes diagnosis from year of uptake period and was categorised into three groups (0–4 years; 5–9 years; 10 years and over).

### Data analysis

Analysis was carried out in Stata version 13 for windows (StataCorp, College Station, TX). Descriptive statistics were used to summarise characteristics of people with diabetes and were stratified according to outcome group. Uptake was calculated as the number of people who participated in the programme (consented *and* attended) and reported as a proportion of the total who were registered. Group specific differences in categorical variables were analysed using Pearson’s chi-square test. The mean and standard deviation were reported if continuous data conformed to normality and the student t-test was conducted to compare mean differences. If data were skewed, the median with associated lower and upper quartile values was reported and the Kruskal Wallis test was utilised. Associations between predictor variables and programme outcomes were examined with multivariable Poisson regression. Adjusted incident rate ratios (IRR) with 95% CI were generated as a measure of association. Predictor variables were selected based on whether they had been reported in the literature as significant predictors of uptake to diabetic retinopathy screening.

### Ethical considerations

Ethical approval for the study was obtained from the Clinical Research Ethics Committee for the Cork Teaching Hospitals (ECM 4 (o)). Patient consent for the use of their medical records was waived by the ethics committee as no patient records or identifiable data were removed from primary care centres. MT acted as a ‘Data processor’ on behalf of the general practitioner and a ‘Data Protection and Confidentiality Agreement’ was signed by the general practitioner and MT.

## Results

### Uptake of Diabetic RetinaScreen

**Figure 3.**
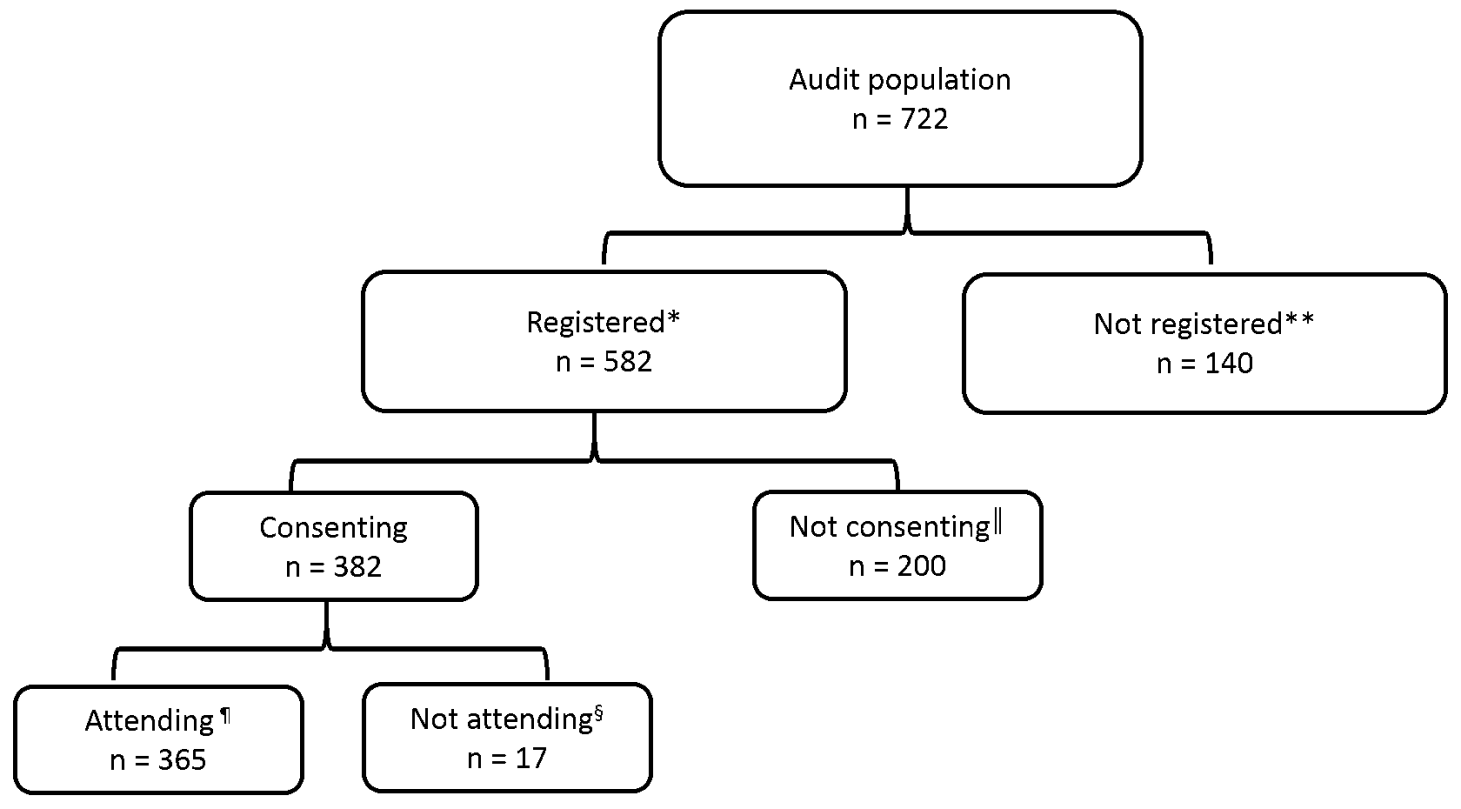
Outcome status of individuals identified during data collection. *Registered, details listed on the screening programme database; invitation letter to avail of screening sent. **Not registered, details not listed on the screening programme database. ^║^Not consenting, did not response to invitation letters; ^¶^Attending, attended screening appointment. ^§^Not attending, responded to invitation letter but did not attend screening appointment.

A total of 722 people with type 1 and type 2 diabetes were identified during data collection ( Figure 3). At the time of data collection, one fifth (n = 140) were not registered with RetinaScreen. A total of 582 people were registered and had been invited to participate in the screening programme. Of these, 66% consented to take part (n = 382), the majority of whom attended screening. Overall, 63% of those who were registered (n = 365), participated; i.e., consented *and* attended ( Figure 3).

Most of the 217 who had not participated in the programme had not consented for the programme to hold and use their details (n = 200, 92%). While the uptake of RetinaScreen was 63% among those who were registered for the programme (n = 365/582), only half (n = 365/722) of the eligible population of people with diabetes had participated in the new national programme at the time of the study.

### Characteristics of the target population

The characteristics of the 582 people who were registered with RetinaScreen are shown in Table 1. The mean age of patients was 63.0 years (SD 13.8), 61% were male and 91% had type 2 diabetes. Approximately half of the sample had evidence of attending existing retinopathy screening services in their medical record (52%).

**Table 1.** Characteristics of the sample who were registered, stratified by outcome status (n = 582).

	Registered (n = 582)	Consenting (n = 381)		Not consenting (n = 200)
Variable	Overall (n = 582)	Attending (n = 365)	Not attending (n = 17)	
	N (%)	N (%)	N (%)	N (%)
**Demographics** **Age years (mean, SD)**	63.0 (13.8)	63.8 (12.8)	57.9 (17.6)	62.1 (15.1)
**Gender [Male]**	355 (61)	227 (62)	6 (35)	122 (61)
**Medical card**	406 (70)	252 (69)	12 (71)	142 (71)
**Diabetes**				
**Type 2**	531 (91)	341 (93)	14 (82)	176 (88)
**Year of diagnosis ≤**2012	533 (92)	337 (92)	14 (82)	182 (91)
**Years since diagnosis (median, IQR)**	8 (5–13)	8 (5–13)	6 (4–10)	9 (5–15)
**Complications** Microvascular Macrovascular	147 (25) 75 (13)	86 (24) 47 (13)	5 (29) 2 (12)	56 (28) 26 (13)
**Hypertension**	294 (50)	189 (52)	7 (41)	91 (46)
Screening history * None Previous attendance	280 (48) 301 (52)	214 (59) 151 (41)	5 (29) 12 (71)	61 (31) 138 (69)

*Evidence of retinopathy screening at existing screening provider in medical record

### Predictors of consenting to Diabetic RetinaScreen

Most people who consented to participate in the programme subsequently attended (n = 365/382, 95.6%). Therefore, consent to be invited to participate was the outcome of interest for the regression analysis. Table 2 presents the results from the Poisson regression analyses. Multivariable analysis indicated that people who had previously attended an existing retinopathy screening service (IRR = 0.65 [95% CI 0.5-0.8]; p<0.001) were less likely to consent.

**Table 2.** Contextual predictors of consenting to RetinaScreen.

Variables	Crude IRR (95% CI) (n=582)	*p*	Adjusted ^1^ IRR (95% CI) (n=582)	*p*
**Demographics** ***Age*** 18–39 years 40–64 years 65+ years ***Gender*** Male Female ***Healthcare cover*** Medical card Private	1 (ref) 1.7 (0.9-3) 1.6 (0.8-2.8) 1 (ref) 1.03 (0.82-1.3) 1 (ref) 0.9 (0.8-1.2)	0.10 0.13 0.68 0.77	1 (ref) 1.4 (0.7-2.7) 1.3 (0.6-2.6) 1 (ref) 0.9 (0.8-1.2) 1 (ref) 1.04 (0.8-1.3)	0.34 0.46 0.69 0.72
**Medical factors** ***Diabetes type*** Type 1 Type 2 ***Years since diagnosis*** 0–4 years 5–9 years 10 + years	1 (ref) 1.4 (0.9-2.1) 1 (ref) 0.9 (0.8-1.3) 0.9 (0.7-1.2)	0.14 0.89 0.80	1 (ref) 1.3 (0.8-1.3) 1 (ref) 0.9 (0.7-1.3) 1.03 (0.8-1.4)	0.28 0.89 0.81
***Attendance to existing retinopathy screening*** None Existing	1 (ref) 0.65 (0.5-0.8)	<0.001	1 (ref) 0.65 (0.5-0.8)	<0.001

^1^variables entered into model: age, gender, healthcare cover, diabetes type, years since diagnosis, screening history

## Discussion

This study outlines uptake of DRS among people with diabetes in Ireland during the initial implementation of a new national screening programme. Over the 14-month period the overall uptake (consenting *and* attending) among people who were registered was 63%. This is similar to the most recent figures (61%) available from RetinaScreen; i.e., people sent a consent letter who attended ^20^, and higher than previously reported in some regional community-based screening initiatives ^3, 4, 6^. Consent was the outcome of interest as this is the first point of engagement with the programme before a patient can attend screening. Over one third of people eligible to participate in RetinaScreen had not consented, suggesting barriers may occur at this stage. Encouragingly, once consented, most people (96%, n = 365/382) attended their screening appointment.

National figures indicate that, in the first round of screening (March 2013 to December 2014), of the 134,513 people who were invited to participate (sent a consent letter), 57.1% consented to RetinaScreen ^18^. This is lower than the proportion of people reported in the current study (66%). While previous studies have found factors such as age ^9, 11^, type of diabetes and duration ^9^ to be associated with DRS uptake, our analysis only found that previous attendance to an existing retinopathy screening service was significantly associated with non-consent. We may expect that people who already are aware of, and familiar with, DRS would be more inclined to attend the new national programme. A lack of awareness of DR and the risk has previously been reported as a barrier to attendance in the international literature ^6, 17, 21– 23^. An Irish study conducted in 2015 which surveyed people with diabetes attending general practices and diabetes outpatient clinics about screening behaviours, reported 91% had never previously heard of RetinaScreen ^24^. However, since then the programme has introduced further advertising and may be more familiar to people with diabetes. Conversely, those attending another screening service may find it is more convenient. RetinaScreen appointments are offered on weekdays and during the working day. It is possible that people may find it difficult to attend appointments at these times. Our follow-up qualitative work with patients indicated competing demands, including work and family commitments were barrier to attendance. Similarly, a 2016 systematic review identified several individual, social, cultural and environmental barriers DRS attendance, including work commitments (e.g. finding it hard to take time off work) ^21^.

We found one fifth of people with diabetes were not registered with RetinaScreen at the time of the study. The introduction of the Cycle of Care, in 2015, may improve RetinaScreen registration rates for those with a medical card as it provides financial remuneration to General Practitioners (GPs) for providing structured care for people with type 2 diabetes who have a medical card. The structured review visit includes monitoring of key processes of care including screening attendance. In this study, 34% of those not registered would not be eligible for the Cycle of Care. Systems should be put in place to support professionals to register and encourage attendance among *all* people with diabetes. With routine management of type 2 diabetes taking place in the community, primary care professionals are well positioned to promote DRS attendance. A recommendation to attend screening from a primary care professional has been found to motivate attendance ^17, 21, 25^. In a survey of GPs in Ireland, 56% identified the time required to register patients as a barrier ^24^. Since this study, RetinaScreen have introduced a number of measures to facilitate registration and consent, including an online referral system (2015), and a single step registration and consent form which can be returned by people with diabetes directly to RetinaScreen (2019) ^19, 26^. It is important to recognise that service innovations evolve as they become more embedded in everyday practice. As such, with new implementation strategies RetinaScreen may have addressed initial challenges and reasons for non-participation may change over time.

It is important to acknowledge the limitations of this study. First, as mentioned, the study was undertaken during the initial phase of an on-going implementation process. However, estimates from the current study are in line with more recent figures from the programme. People may be attending private providers, and we cannot assess this using the current data. Quantitative data were extracted using a standardised extraction template and relevant quality checks were applied to the data. We acknowledge that the completeness and accuracy of our study is dependent on the consistency and timely application of codes in each practice. However, both practices have systems in place to ensure that databases are maintained to a high standard. The type of predictors examined by this study are limited to those available in patient records. Unfortunately the data did not include several factors which have consistently been found to be important in previous studies, for example, socio economic status (SES) ^27– 30^, self-management, and history of glycaemic control ^17^, psycho-social factors (e.g. attitudes, beliefs and knowledge about DRS ^6, 17, 21– 23^ and recommendations from a health care professional ^17, 21, 22, 25^.

### Recommendations for future research

Given the limited nature of the data a consideration for future research could be to replicate this study using more extensive audit data. Data is routinely collected from practices participating in diabetes care initiatives across Ireland, for example, the Midlands Diabetes Structured Care Programme which reported on RetinaScreen uptake in the most recent audit ^3^. Determining how to enhance the uptake of DRS is recognised as an important implementation challenge for health systems, as evidenced by dedicated research programmes in the UK ^14, 21^, Canada ^8, 31^, and Australia ^32^. Qualitative work with Irish people with diabetes and health care professionals has been conducted to explore barriers and enablers of DRS uptake and to inform the development an intervention to be delivered in general practice ^33^. The feasibility trial of this intervention is currently underway ^34^.

## Conclusion

We found over one third of people eligible to participate in the free national retinal screening programme, Diabetic RetinaScreen, had not done so. The results suggest DRS attendance could be supported by raising awareness of screening and supporting professionals to register and encourage their patients with diabetes to attend. Type 2 diabetes, which accounts for about 90% of all cases of diabetes ^35^, is largely managed in primary care, making this a suitable setting in which to introduce strategies to support DRS uptake. Further research is needed to better understand barriers and enablers of DRS uptake in the Irish context, and to determine strategies would effectively target these factors. In Ireland, the population eligible for screening is increasing each year ^36^, therefore implementing effective strategies to maximise uptake of DRS must be a priority from the outset.

## Data availability

Permission was not sought from participating practices or the Clinical Research Ethics Committee to share the data outside of the research team. De-identified data from the current study are available for further (collaborative) research purposes on reasonable request. Available datasets include the audit data. To access the data, please contact the corresponding author ( fiona.riordan@ucc.ie) or the Principal Investigator ( patricia.kearney@ucc.ie). Researchers must provide a written proposal on how the data will be used in research before access is granted.

## Ethics

The research was approved in Ireland by the Clinical Research Ethics Committee of the Cork Teaching Hospitals, UCC.
