# Supplementary material for: Understanding the uptake of a national retinopathy screening programme: An audit of people with diabetes in two large primary care centres
Source: HRB Open Res. 2019 Nov 27;2:17. Originally published 2019 Jul 26. [Version 2] doi: 10.12688/hrbopenres.12926.2 (PMC7016880; doi:10.12688/hrbopenres.12926.2)
Supplement: Peer review history

# Peer review: Reviewer response for version 2

**DOI:** 10.21956/hrbopenres.14051.r26963

**Published:** 2019-12-04

**Authors:** Irene M. Stratton

**Affiliations:** 1 Gloucestershire Retinal Research Group, Cheltenham General Hospital, Cheltenham, UK

Thank you for your careful responses to my questions and the changes you have made to the paper.

 My main concern here is with the prominence given to the "8.2%" with diabetic retinopathy. Self-report is notoriously poor in the area of ophthalmology. I sit in a screening programme office for at least half a day a week and have done so for the past decade. Many of those attending for screening have no idea why they are having their eyes photographed and don't understand the report letters and so would not know if they had DR or not - especially if it was microaneurysms alone.

 I am happy with the rest of the paper but this number should be relegated to the discussion and more reliable estimates of the prevalence of diabetic retinopathy provided.

I confirm that I have read this submission and believe that I have an appropriate level of expertise to confirm that it is of an acceptable scientific standard, however I have significant reservations, as outlined above.

# Response

**Published:** 2019-12-10

**Authors:** Fiona Riordan

**Affiliations:** University College Cork, Ireland

Thank you for your review. We have revised the introduction to place the emphasis on objective prevalence estimates. We have moved the self-report estimate to the discussion as suggested.

# Peer review: Reviewer response for version 1

**DOI:** 10.21956/hrbopenres.14005.r26814

**Published:** 2019-10-14

**Authors:** Irene M. Stratton

**Affiliations:** 1 Gloucestershire Retinal Research Group, Cheltenham General Hospital, Cheltenham, UK

"Diabetic retinopathy (DR) affects 8.2% of the Irish population with type 2 diabetes over 50 years and is one of the leading causes of blindness among working-age adults". Diabetic retinopathy affects about 30% of people in England with diabetes and similar in Scotland so it's unlikely to be 8.2% in Ireland. That's any DR (one microaneurysm or more). If the authors mean sight threatening DR then this should be stated. 

 "Screening uptake has also been identified as a challenge in countries such as the UK; with attendance rates ranging from 56–90% ^7^". This is in Wales - is this range between GP surgeries or areas of Wales?

 Are the appointments fixed time appointments? Are the patients able to change the time or place of the screening? Are the appointments weekdays and working day only? 

 Maybe those who were recorded as already having attended screening thought that the service they had been using was as good (maybe more convenient) than the DRS? 

 Barriers to consent would seem to me to be a bigger problem than attendance.

I confirm that I have read this submission and believe that I have an appropriate level of expertise to confirm that it is of an acceptable scientific standard, however I have significant reservations, as outlined above.

# Response

**Published:** 2019-11-08

**Authors:** Fiona Riordan

**Affiliations:** University College Cork, Ireland

Thank you for your feedback. We have detailed our response to each comment below.

**"Diabetic retinopathy (DR) affects 8.2% of the Irish population with type 2 diabetes over 50 years and is one of the leading causes of blindness among working-age adults". Diabetic retinopathy affects about 30% of people in England with diabetes and similar in Scotland so it's unlikely to be 8.2% in Ireland. That's any DR (one microaneurysm or more). If the authors mean sight threatening DR then this should be stated.**

8.2% is the prevalence based on self-report among a cohort aged 50 and over with type 2 only who participated in The Irish Longitudinal Study of Ageing (TILDA).  Were the whole population to be taken into account including <50 years and people with type 1 this figure would be closer to 30%. In TILDA retinopathy was confirmed using the question ‘Has a doctor ever told you that you have any of the following conditions related to your diabetes?’. One of the listed conditions was ‘damage to the back of your eye (diabetic retinopathy)’. In the Irish prevalence study, it was clarified that the prevalence of retinopathy was much lower than that reported by a previous study, The Cost of Diabetes in Ireland Study (CODEIRE) (retinopathy: 15% vs. 8.2%), outlining that this may be explained by differences in data collection methods (medical records vs. self-report). A regional study involving primary care patients (type 1 and type 2, and based on objective data), found a higher prevalence of 24.8%, similar to an estimate in a comparable cohort in a different region (25.6%).

We have amended the background section as follows (page 3, line 53):

*Diabetic retinopathy (DR) is the most common microvascular complication of diabetes. DR affects 8.2% of the Irish population over 50 years with type 2 diabetes (1), and is the leading causes of blindness among adults of working age* *(2). This estimate is based on self-report; studies among regional cohorts of primary care patients with type 1 and 2, based on objective data, have reported higher estimates (25-26%) (3, 4).*

**"Screening uptake has also been identified as a challenge in countries such as the UK; with attendance rates ranging from 56–90%** ^**7**^ **". This is in Wales - is this range between GP surgeries or areas of Wales?**

There should have been an additional reference here (Lawrenson *et al.*) citing range in the uptake rates across England.  We have revised this line (61) to include reference to studies which illustrate the variation in uptake rates internationally. The Welsh paper by Thomas *et al.* reports the uptake of the Diabetic Retinopathy Screening Service for Wales (DRSSW) as 80%. This figure is included in the range now provided (page 3 line 64):

*Screening uptake has also been identified as a challenge internationally, with attendance rates ranging from 28-92% (8-14).*

**Are the appointments fixed time appointments? Are the patients able to change the time or place of the screening? Are the appointments weekdays and working day only?**

People can contact RetinaScreen to change the time of the appointments. They cannot change the location; the appointment is offered for their local screening centre. Appointments are weekdays and during working day only. As part of a process to develop an intervention to improve the uptake of retinopathy screening (now cited in the ‘Recommendations for future research’ section on page 11) we conducted interviews and consensus meetings with people with diabetes. They reported difficulties arranging appointments during the day. We have clarified this point about rescheduling appointments in the methods section (page 4, line 87):

*Once consented they receive an appointment for a fixed time in their local screening centre. They can contact RetinaScreen to change the time and date of their appointment*.

We have also reflected on this in the discussion section (page 9, 216 -226):

An Irish study conducted in 2015 which surveyed people with diabetes attending general practices and diabetes outpatient clinics about screening behaviours, reported 91% had never previously heard of RetinaScreen (30). *However, since then the programme has introduced further advertising and may be more familiar to people with diabetes. Conversely, those attending another screening service may find it is more convenient.* *RetinaScreen appointments are offered on weekdays and during the working day. It is possible that people may find it difficult to attend appointments at these times. Our follow-up qualitative work with patients indicated competing demands, including work and family commitments were barrier to attendance. Similarly, a 2016 systematic review identified several individual, social, cultural and environmental barriers DRS attendance, including work commitments (e.g. finding it hard to take time off work)* *(25).*

**Maybe those who were recorded as already having attended screening thought that the service they had been using was as good (maybe more convenient) than the DRS?** 

Yes, this could be the case. People who we interviewed as part of our follow-up qualitative work  were influenced by the familiarity or locality of an existing service. There was also confusion with regard to the service being offered by the national programme, RetinaScreen, and other providers. For example, some people were attending an optician and thought this was equivalent to attending the screening programme. RetinaScreen is quality assured and facilitates patients to access the appropriate treatment pathway should they require further treatment. Therefore, it is important patients participate in the programme. We have included a line to clarify that people may find existing service more convenient (page 9, 216 -226) which links in with the point above in relation to appointment times:

*Conversely, those attending another screening service may find  it is more convenient.* *RetinaScreen appointments are offered on weekdays and during the working day. It is possible that people may find it difficult to attend appointments at these times. Our follow-up qualitative work with patients indicated competing demands, including work and family commitments were barrier to attendance.*

**Barriers to consent would seem to me to be a bigger problem than attendance.**

We agree. Providing consent is the first point at which people are required to interact with the programme. Therefore, as we would expect the main barriers occur this point, when people decide whether to participate in the programme. We have included a line to clarify this in the discussion and abstract:

Discussion (page 8, line 208)

*Over one third of people eligible to participate in RetinaScreen had not consented, suggesting barriers may occur at this stage. Encouragingly, once consented, most people (96%, n = 365/382) attended their screening appointment.*  

Abstract (page 2, line 45):

*Implementing strategies to improve DRS uptake, in particular barriers to consent, should be a priority.*

# Peer review: Reviewer response for version 1

**DOI:** 10.21956/hrbopenres.14005.r26815

**Published:** 2019-10-08

**Authors:** Amelia J. Lake

**Affiliations:** 1 School of Psychology, Deakin University, Geelong, Vic, Australia; 2 The Australian Centre for Behavioural Research in Diabetes, Diabetes Victoria, Melbourne, Australia

The study described in this paper is rigorously designed and conducted and an appropriate early step to optimise uptake of RetinaScreen, a nationally-coordinated retinal screening programme in Ireland.

 The paper describes an audit of RetinaScreen registration, consent and uptake for adults living with diabetes from two primary care clinics. The audit was conducted during initial implementation (Nov 2013 – Aug 2015).

 Of the audit population (N=722), 582 had been registered with RetinaScreen. Of those, 382 consented to be part of the RetinaScreen programme with 365 attending a retinal screening appointment.

 With such high uptake for those who consented, the aim of the study was to identifying factors associated with non-consent to participate in RetinaScreen.

 The authors reported that the only variable significantly associated with non-consent was whether people had previously attended another retinal screening service. This is a useful finding which highlights the importance of promoting ease and benefits of RetinaScreen to both customers and operators of existing private optometry practices.

 I enjoyed reading the paper and the study is scientifically sound. I note the following points for consideration by the authors: 

**Is the work clearly and accurately presented and does it cite the current literature?**

 The work is clearly and accurately presented. However, many of the references cited are specific to the Irish context and/or reflect the time preceding and during the study period (i.e. <2016). The subject matter is relevant internationally and I suggest that the authors consider several reviews and studies (noted below) when discussing both known barriers and directions for future research.

**Are the conclusions drawn adequately supported by the results?**

 Although the authors discussed the limited nature of the source data, I suggest that they more strongly emphasise this point. Existing research has consistently demonstrated that demographic (e.g. socioeconomic status, ethnicity), clinical (e.g. sub-optimal glycaemic management) and psycho-social (e.g attitudes, beliefs, knowledge, healthcare professional recommendation, confusion between routine eye care and retinal screening) factors also strongly influence uptake and engagement with retinal screening programmes. Unfortunately, none of these factors could be considered in the current study.

**Other points:**

 As noted by the authors, this work was conducted during an initial implementation phase and service innovation has evolved since the audit data was collected. As such, the paper would benefit from inclusion of a 'Recommendations for future research' section. Suggestions include: replication of this study (with a greater breadth of audit data) to revisit factors associated with non-consent; exploration of individual-level and practice-level barriers to RetinaScreen registration and consent to participate.If possible, the authors may like to comment on any work currently being undertaken in this area.

**Minor citation, grammatical and formatting errors:  **

- I commend the authors’ use of person-centered language, which is in accordance with international practice (see diabetes language position statements published by Diabetes Australia, Diabetes UK and the American Diabetes Association). I suggest that the authors replace ‘patient’ with appropriate terminology throughout the paper.
- Please rectify typo in paragraph 1 of Methods (‘add’ repeated in the one sentence).
- Please amend disparity between the number of people with diabetes registered with RetinaScreen reported in-text (Methods para.1, n=141) and in Fig.3 (n=140).
- Please include RetinaScreen (2019) citation and URL in references (see ‘single step registration and consent’ in Discussion) and include URL where appropriate (e.g. reference number 14, 16, 26).

In conclusion, the article is scientifically valid in it’s current form and I approve it for indexing. The issues that I have raised pertain to restrictions on the conclusions that can be drawn, given the limited data on which the analyses are based. Nonetheless, the findings are very interesting and will assist retinal screening programme implementers in determining where to direct promotional messaging and strategies, both nationally and internationally.

I confirm that I have read this submission and believe that I have an appropriate level of expertise to confirm that it is of an acceptable scientific standard.

## References

[1] Diabetic Retinopathy Screening: A Systematic Review on Patients’ Non-Attendance. *International Journal of Environmental Research and Public Health*.2018;15(1) : 10.3390/ijerph15010157 10.3390/ijerph15010157 PMC580025629351207

[2] : Systematic review on barriers and enablers for access to diabetic retinopathy screening services in different income settings. *PLoS One*.2019;14(4) : 10.1371/journal.pone.0198979 e0198979 10.1371/journal.pone.0198979 31013274PMC6478270

[3] : Screening attendance, age group and diabetic retinopathy level at first screen. *Diabet Med*.33(7) : 10.1111/dme.12957 904-11 10.1111/dme.12957 26337435PMC5057362

# Response

**Published:** 2019-11-08

**Authors:** Fiona Riordan

**Affiliations:** University College Cork, Ireland

Thank you for your comments and feedback and for the suggested references. We have referenced these studies in the discussion:

(page 9, line 217 - 228)

*A lack of awareness of DR and the risk has previously been reported as a barrier to attendance in the international literature (6, 18, 24-27).*

(page 9, line 238 - 242)

*In several international studies, a recommendation to attend screening from a  primary care professional has been found to motivate DRS attendance* *(24-26, 29)* *. A recent systematic review of barriers and enablers to DRS across different income groups cited poor patient-physician communication as a key barrier to DRS attendance.(27)*

**Are the conclusions drawn adequately supported by the results?**

**Although the authors discussed the limited nature of the source data, I suggest that they more strongly emphasise this point. Existing research has consistently demonstrated that demographic (e.g. socioeconomic status, ethnicity), clinical (e.g. sub-optimal glycaemic management) and psycho-social (e.g attitudes, beliefs, knowledge, healthcare professional recommendation, confusion between routine eye care and retinal screening) factors also strongly influence uptake and engagement with retinal screening programmes. Unfortunately, none of these factors could be considered in the current study.**

We have revised the limitations section to emphasise these other factors (page 10, line 258 – 262):

*Unfortunately the data did not include several important factors which have consistently* *been found to be important in previous studies, for example, socio economic status (SES) (30-33), self-management, and history of glycaemic control (24), and psycho-social factors (e.g. attitudes, beliefs and knowledge about DRS (6, 18, 24-27) and recommendations from a health care professional* *(24-26, 29)* *).*

**Other points:**

**As noted by the authors, this work was conducted during an initial implementation phase and service innovation has evolved since the audit data was collected. As such, the paper would benefit from inclusion of a 'Recommendations for future research' section. Suggestions include: replication of this study (with a greater breadth of audit data) to revisit factors associated with non-consent; exploration of individual-level and practice-level barriers to RetinaScreen registration and consent to participate.If possible, the authors may like to comment on any work currently being undertaken in this area.**

We have included the following section (page 11, line 264 -273) on future research with reference to studies in the UK, Canada and Australia:

***Recommendations for future research***

*Given the limited nature of the data a consideration for future research could be to replicate this study using more extensive audit data.  Data is routinely collected from practices participating in diabetes care initiatives across Ireland, for example, the Midlands Diabetes Structured Care Programme  which reported on RetinaScreen uptake in the most recent audit (22).* *Determining how to enhance the uptake of DRS is recognised as an important implementation challenge for health systems, as evidenced by dedicated research programmes in the UK (13, 25), Canada (34, 35), and Australia (36). Qualitative work with Irish people with diabetes and health care professionals has been conducted to explore barriers and enablers of DRS uptake and to inform the development an intervention to be delivered in general practice (37). The feasibility trial of this intervention is currently underway (38).*

**Minor citation, grammatical and formatting errors:**

**I commend the authors’ use of person-centered language, which is in accordance with international practice (see diabetes language position statements published by Diabetes Australia, Diabetes UK and the American Diabetes Association). I suggest that the authors replace ‘patient’ with appropriate terminology throughout the paper.**

We have amended this throughout.

**Please rectify typo in paragraph 1 of Methods (‘add’ repeated in the one sentence).**

Amended.

**Please amend disparity between the number of people with diabetes registered with RetinaScreen reported in-text (Methods para.1, n=141) and in Fig.3 (n=140).**

Amended; 140 is correct.

**Please include RetinaScreen (2019) citation and URL in references (see ‘single step registration and consent’ in Discussion) and include URL where appropriate (e.g. reference number 14, 16, 26).**

These references have been added in appropriate location (line 57, 78, 84, 242)
